# Tetraspanin CD9: A Key Regulator of Cell Adhesion in the Immune System

**DOI:** 10.3389/fimmu.2018.00863

**Published:** 2018-04-30

**Authors:** Raquel Reyes, Beatriz Cardeñes, Yesenia Machado-Pineda, Carlos Cabañas

**Affiliations:** ^1^Departamento de Biología Celular e Inmunología, Centro de Biología Molecular Severo Ochoa (CSIC-UAM), Madrid, Spain; ^2^Departamento de Inmunología, Oftalmología y OTR (IO2), Facultad de Medicina, Universidad Complutense, Madrid, Spain

**Keywords:** CD9, tetraspanins, integrins, ICAM1, activated leukocyte cell adhesion molecule, ADAM17, lymphocyte function-associated antigen 1, very late activation antigen 4

## Abstract

The tetraspanin CD9 is expressed by all the major subsets of leukocytes (B cells, CD4^+^ T cells, CD8^+^ T cells, natural killer cells, granulocytes, monocytes and macrophages, and immature and mature dendritic cells) and also at a high level by endothelial cells. As a typical member of the tetraspanin superfamily, a prominent feature of CD9 is its propensity to engage in a multitude of interactions with other tetraspanins as well as with different transmembrane and intracellular proteins within the context of defined membranal domains termed tetraspanin-enriched microdomains (TEMs). Through these associations, CD9 influences many cellular activities in the different subtypes of leukocytes and in endothelial cells, including intracellular signaling, proliferation, activation, survival, migration, invasion, adhesion, and diapedesis. Several excellent reviews have already covered the topic of how tetraspanins, including CD9, regulate these cellular processes in the different cells of the immune system. In this mini-review, however, we will focus particularly on describing and discussing the regulatory effects exerted by CD9 on different adhesion molecules that play pivotal roles in the physiology of leukocytes and endothelial cells, with a particular emphasis in the regulation of adhesion molecules of the integrin and immunoglobulin superfamilies.

## Introduction

CD9, or Tspan 29 in the systematic nomenclature, is a 21–24 kDa member of the tetraspanin protein family. Tetraspanins are structurally characterized by containing four transmembrane domains, which delimit a small extracellular loop (known as SEL or EC1), a large extracellular loop (termed LEL or EC2), and short intracellular N- and C-terminal tails. Within the LEL domain five α-helices (A-E) can be distinguished, with helices A, B, and E defining a region well conserved among different members (“constant region”) that is involved in tetraspanin dimerization and oligomerization, whereas helices C and D define the “variable region” of the LEL, involved in most lateral interactions of tetraspanins with other membrane proteins. The presence of a CCG motif after the B helix in the LEL domain, with its two cysteines engaged in the formation of intradomain disulfide bonds with other conserved cysteines, membrane-proximal palmitoylations, and hydrophilic residues within transmembrane domains are also defining features of tetraspanin members. Tetraspanins are glycoproteins which generally contain several N-glycosylation sites in the LEL domain (1), and in this regard, CD9 is peculiar as it contains only one N-glycosylation site that is located in its SEL domain (2).

CD9 shows a wide cellular and tissue distribution and was initially identified as a lymphohematopoietic marker (3) and then implicated in a range of cellular functions, including motility, proliferation, differentiation, fusion, and adhesion [reviewed in Ref. (1, 4–6)]. Not surprinsingly, given its involvement in such a range of crucial cell activities, CD9 plays a major role in critical physiological and pathological processes, including sperm–egg fusion, neurite outgrowth, myotube formation, viral infections, tumorigenicity, and metastasis [reviewed in Ref. (7–9)]. As for other members of the tetraspanin family, the biological functions of CD9 greatly depend on the multitude of dynamic interactions that this molecule is able to establish with other transmembrane and cytoplasmic proteins within the context of a specific type of membrane domains, called tetraspanin-enriched microdomains (TEMs) (10, 11). CD9 in TEMs can thus affect, either directly or indirectly, the activity of numerous transmembrane and intracellular proteins such as metalloproteinases and other enzymes, ion channels, receptors for growth factors, cytokines and chemokines, transporters, signaling transducers, and cytoskeletal linkers (Table 1). CD9 can potentially alter the activity of these molecules through different mechanisms including their selec-tive confinement in TEMs or segregation into separate microdomain compartments, which would hinder their access to their cognate substrates or binding of their extracellular or intracellular ligands (10, 11).

**Table 1:** List of molecules reported to associate with CD9.

CD9-associated molecules	Other associated tetraspanins	Reference
**Adhesion molecules**		
Integrins		
α1β1		(12)
α2β1	CD151	(13, 14)
α3β1	CD63, CD81, CD82, CD151, NAG-2, Co-029	(13, 14)
α4β1 [very late activation antigen 4 (VLA-4)]	CD53, CD81, CD82, CD151	(15, 16)
α5β1 (VLA-5)	CD151	(16)
α6β1	CD63, CD81, CD82, CD151, NAG-2, Co-029	(15, 17)
α7β1	CD151	(18)
αIIbβ3	CD151	(19, 20)
α6β4	CD151	(13)
αLβ2 (lymphocyte function-associated antigen 1)	CD81, CD82	(21–23)
**Immunoglobulin-SF members**		
Intercellular adhesion molecule 1 (CD54)	CD151	(24, 25)
EpCAM (CD326)	Co-29, Tspan8, D6.1A	(26)
B-CAM/Lu (CD239)		(27)
Activated leukocyte cell adhesion molecule (cD166)		(28, 29)
Vascular cell adhesion molecule-1 (CD106)	CD151	(24)
**Other adhesion receptors**		
CD42		(20, 30)
CD44	Co-29, Tspan8, D6.1A	(13)
CD47		(20)
Claudin-1		(31)
Syndecan		(13)

**Immune system molecules**		
HLA-DR	CD153, CD81, CD82	(15)
CD2	CD53	(32)
CD3	CD81, CD82	(33)
CD4	CD81, CD82	(33)
CD5		(33)
CD19	CD81	(34)
CD46		(12)

**Growth factors**		
Pro-TGF-α		(35)
Pro-HB-EGF		(36)

**Signaling molecules**		
CD117	CD63, CD81	(37)
GPCR56	CD81	(38)
PI4K	CD81, CD151, CD63	(39)
PKC	CD53, CD81, CD82, CD151	(27, 40)

**Other proteins**		
ADAM2	CD81	(41)
ADAM10	CD81, CD82	(42, 43)
ADAM17		(42, 44)
CD36		(45)
CD26 (DPPIV)		(27, 46)
CD224	CD37, CD81, CD53, CD82	(27)
CTL1/CD92		(27)
CTL2		(27)
EWI-2	CD63, CD81, CD82, CD151	(47)
EWI-F	CD81	(48)
Hem-1		(27)
TADG-15		(27)
Syntaxins 3 and 4A		(27)

It is striking that the basal expression of CD9 in the major types of leukocytes from freshly drawn blood [monocytes, natural killer (NK) cells, B cells, CD8^+^ T cells, and CD4^+^ T cells] is usually low (49, 50). However, its expression in these leukocyte subpopulations increases following their culture, being particularly high in monocytes (8, 49, 50). Likewise, CD9 is also clearly detected in activated lymphoblasts derived from PHA/interleukin-2-stimulated PBMCs (21). CD9 is also expressed by the different subsets of human and murine dendritic cells (DCs), with the exception of mouse plasmacytoid DCs (51). In contrast to the relatively low expression on resting leukocytes, the expression of CD9 is particularly high on endothelial cells (52), in keeping with the crucial role this tetraspanin plays in regulating the firm adhesion and transendothelial migration of leukocytes (24, 25).

Several excellent reviews have already covered the topic of how tetraspanins, including CD9, participate in the regulation of specific functions in different cells of the immune system (4, 8, 53, 54). In this review, however, we will focus particularly on discussing the regulatory effects exerted by CD9 on different adhesion molecules that play pivotal roles in the physiology of leukocytes and endothelial cells, with a particular emphasis in the regulation of adhesion molecules of the integrin and immunoglobulin superfamilies.

## CD9 Regulates Adhesion Molecules at the Immune Synapse (IS) and T Lymphocyte Activation

**Figure 1:**
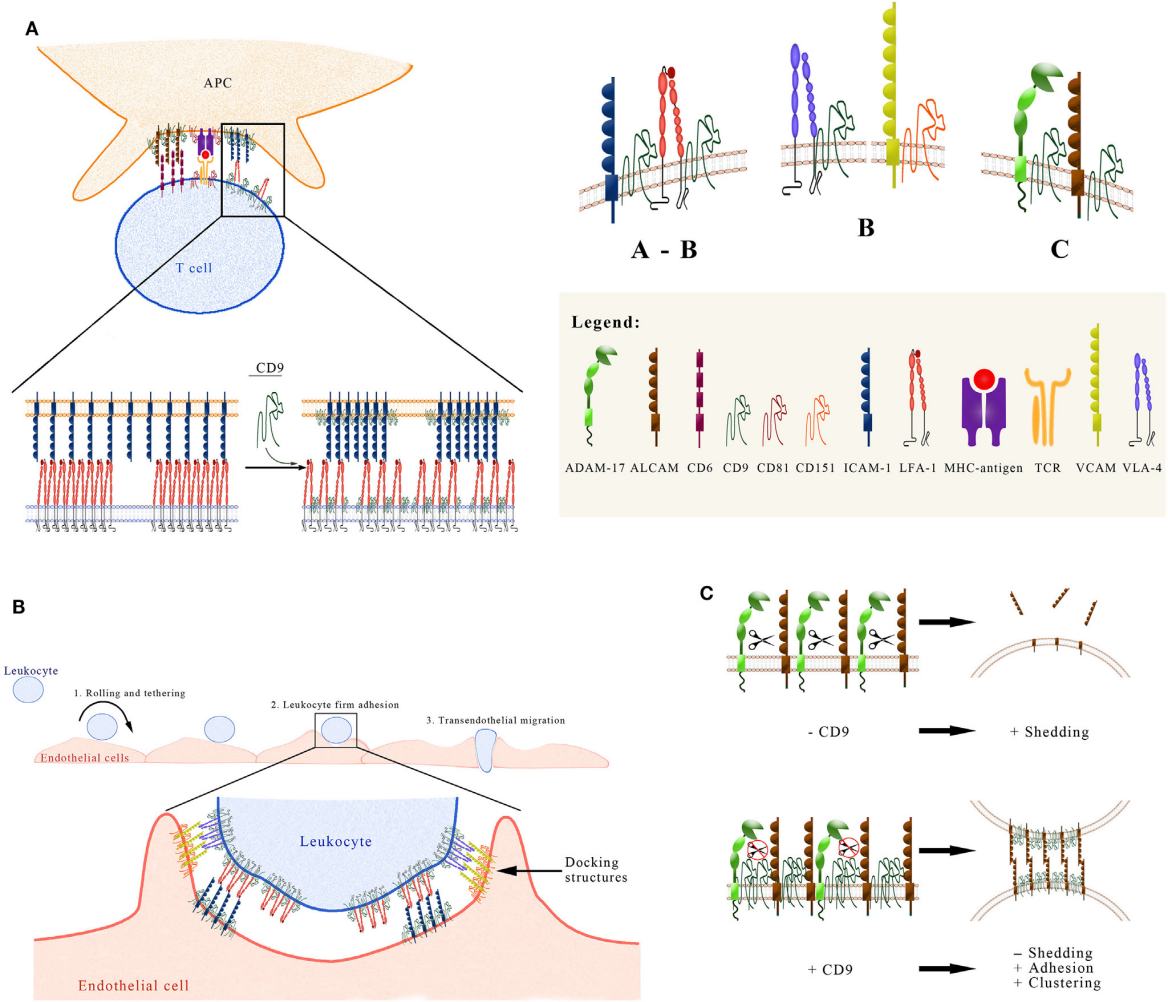
Functional regulation exerted by CD9 on the activity of some immune system adhesion molecules. **(A)** CD9 regulates ICAM and lymphocyte function-associated antigen 1 (LFA-1) at the immune synapse (IS). Interactions between LFA-1 on the T cell, and its ligand intercellular adhesion molecule 1 (ICAM-1) on the APC surface, take place at the peripheral area of the IS (pSMAC) and are crucial for IS formation and stabilization. The tetraspanin CD9 plays an important role in the IS in two different ways: (1) Through its association with LFA-1 on the T cell, CD9 controls the state of aggregation and adhesive capacity of this integrin. Neoexpression/overexpresion of CD9 reduces the integrin adhesive capacity by generating a larger number of clusters of LFA-1 molecules that individually display a smaller size. (2) On the APC surface CD9 recruits ICAM-1 into TEMs, thus increasing its adhesive capacity. **(B)** CD9 regulates leukocyte firm adhesion on endothelial cells. The multi-step paradigm of the leukocyte extravasation cascade includes the initial tethering and rolling of the leukocyte on the endothelial surface, followed by the firm adhesion step and transmigration either between two endothelial cells or through the body of an endothelial cell. The firm adhesion step is mediated by the high-affinity interaction of leukocyte integrins LFA-1 (αLβ2) and Mac-1 (αMβ2) with their endothelial counter-receptor ICAM-1, and of integrin VLA-4 (α4β1) with its endothelial ligand VCAM-1. ICAM-1 and VCAM-1 are preorganized in endothelial adhesive platforms (EAPs), through their association with CD9 and CD151 respectively. After leukocyte binding, EAPs evolve into three-dimensional docking structures that emanate from the endothelial surface and embrace the leukocyte. **(C)** CD9 affects the shedding of leukocyte adhesion molecules mediated by ADAM17. The recruitment of ADAM17 into CD9-organized TEMs (low panel), following the overexpression or neoexpression of this tetraspanin, exerts a negative regulation on the sheddase activity of ADAM17 against different substrates on leukocytic cells, including activated leukocyte cell adhesion molecule (ALCAM). This negative regulation on ADAM17 activity accounts for an increased expression of ALCAM on the cell surface. Additionally, CD9 also induces the aggregation of ALCAM and the concomitant increase in its avidity. Therefore, CD9 augments ALCAM-mediated cell–cell adhesion through this dual mechanism.

Recognition of antigenic-peptides bound to MCH-I and MCH-II molecules on the surface of antigen presenting cells (APCs) is essential for activation of CD8^+^ and CD4^+^ T cells, respectively, and triggers the initiation of T cell-mediated immune responses. This process requires the establishment of a dynamic structure at the APC-T cell contact area, termed as IS. In its mature form, the IS contains a clearly distinguishable central region (cSMAC), where clusters of TcR/CD3, costimulatory and signaling molecules concentrate, and a peripheral region (pSMAC) highly enriched in integrins and other adhesion molecules (55, 56). The establishment of IS proceeds through a series of spatiotemporal segregated events, including the initial scanning of antigen peptide-loaded MHC molecules, the specific recognition of antigen-loaded MHC, and signaling by the TcR/CD3 complex and stabilization of the IS. The interaction of different adhesion receptors on either side of the IS (i.e., on the APC or on the T cell) with their specific ligands on the opposing side is essential during the initial scanning and stabilization of the IS. In particular, interactions of integrin lymphocyte function-associated antigen 1 (LFA-1) (αLβ2) on the T cell with its ligands intercellular adhesion molecule 1 (ICAM-1) and ICAM-3 on the APC surface are crucial for IS formation and stabilization. During the initial scanning of MHC-peptide complexes on the surface of APCs, the affinity of T cell LFA-1 for its ligands is low/intermediate, but inside-out signaling triggered from the TcR/CD3 complex upon recognition of the cognate MHC-peptide induces the high-affinity state of this integrin, leading to strong ligand binding and stabilization of the IS (57, 58). TcR and LFA-1 molecules on immune cells are preorganized in nanoclusters that coalesce into larger aggregates following ligand binding (59–62). CD9 has been shown to engage through its LEL domain in direct interactions with LFA-1 on the surface of different types of leukocytes, including T lymphocytes and monocyte/macrophage-like cells (21). Through its association with LFA-1, CD9 controls the state of aggregation and adhesive capacity of this integrin. Through the use of different strategies, such as monoclonal antibody (mAbs) specific for CD9, as well as genetic approaches based on its silencing or neoexpression, it was demonstrated that CD9 exerts a negative regulation on the adhesive capacity of LFA-1. The mechanism involved in this inhibitory effect of CD9 on LFA-1 activity does not rely upon changes in the integrin affinity state, as inferred from the unaltered expression of the activation-reporter 24 epitope (21). These findings concur with the observation that talin relocalization, which is required for the induction of conformational changes in LFA-1 and acquisition of increased affinity through inside-out signaling [reviewed in Ref. (63)], is not altered in T cells with silenced CD9 and CD151 (64). Thus, the negative regulation exerted by CD9 on the adhesive capacity of LFA-1 is rather related to alterations in its state of aggregation and the control of its binding valency (avidity). Interestingly, confocal and TIRF microscopy analyses showed that expression of CD9 in lymphocytic and monocytic cells induces LFA-1 molecules to become organized in a larger number of clusters but individually displaying a smaller size, which would account for the reduced integrin adhesive capacity (21) (Figure 1A). The association of LFA-1 with the actin cytoskeleton has long been known to control the avidity of this integrin through regulation of its dynamic reorganization into microclusters (65). Thus, although the exact mechanism by which CD9 alters the state of aggregation of LFA-1 molecules has not been resolved, it likely relies upon the linkage of this tetraspanin with the microfilaments of the actin cytoskeleton through the Ezrin–Radixin–Moesin (ERM) proteins (66).

In addition to LFA-1, VLA-4 (α4β1) is another integrin predominantly expressed on hematopoietic cells, which also concentrates at the pSMAC playing a role in the stabilization of the IS and in T cell co-stimulation, though the precise ligand for this integrin on the APC is still unknown (67). The two classic ligands of integrin α4β1, alternatively spliced fibronectin and VCAM-1, are not expressed on APCs. Alternative ligands of this integrin are members of the junctional adhesion molecules (JAM) family, but it has not been demonstrated so far whether these molecules play a role in the IS. Contrary to CD81, which strongly colocalizes with CD3 and contributes to cSMAC formation, becoming segregated from LFA-1 which is mainly found at the pSMAC, CD9 (along with CD151) shows less colocalization with CD3, indicating that it is not involved in the reorganization and clustering of CD3 at the IS (64). In contrast, CD9 does associate with integrin α4β1 on T cells and drives both the accumulation of the high-affinity form of this integrin at the IS and the subsequent downstream signaling (64). CD9 (or CD151) silencing results in diminished relocalization of integrin α4β1 to the IS, reduced accumulation of high-affinity β1 integrins at the cell–cell contact area, and decreased downstream integrin signaling.

Besides their adhesive function, integrins also work as efficient bidirectional signaling molecules (68). In T cells, LFA-1 costimulation lowers the TcR-mediated activation threshold (69, 70). Similarly, ligation of integrin α4β1 also provides costimulatory signaling (71). Downstream signaling from these adhesion receptors is mediated through interactions with a number of adaptor and signaling molecules (72, 73), whose activity, location, and aggregation may be regulated by tetraspanins through their selec-tive inclusion in, or exclusion from, specific TEMs. CD9 and CD151 have been shown to increase integrin-dependent ERK1/2 signaling, and the knockdown of these tetraspanins reduces the phosphorylation of focal adhesion kinase (FAK) and ERK1/2 integrin downstream targets and impairs the enrichment of phosphorylated FAK at the IS (64).

Activated leukocyte cell adhesion molecule (ALCAM) or CD166 is a member of the immunoglobulin superfamily of CAMs that can engage in homophilic (ALCAM–ALCAM) as well as heterophilic (ALCAM–CD6) interactions. ALCAM-mediated adhesion is crucial in different physiological and pathological situations, with particular relevance in immune responses, collective cell migration, cancer metastasis, neuronal development, and leukocyte migration across blood–brain barrier (BBB) in multiple sclerosis and autoimmune encephalomyelitis. ALCAM on APCs engages in heterophilic interactions with CD6 on T cells, with both molecules becoming redistributed to the IS in an antigen-dependent manner, which have been shown to play an important role in the formation and stabilization of the IS (74). They also provide costimulatory signals and thus contribute to T cell activation and proliferation (75–77). ALCAM-mediated APC-T cell adhesion requires upregulation of ALCAM avidity for CD6, which is brought about by the aggregation of ALCAM molecules on the cell surface, a process that is in turn controlled by their dynamic association with the actin cytoskeleton. Such dynamic linkage with the actin cytoskeleton takes place through the interaction of a C-terminal KTEA PDZ-binding motif and a membrane-proximal positively charged stretch in ALCAM’s cytoplasmic tail, with the adaptor proteins syntenin-1 and the ERM protein ezrin, respectively (28, 78).

In this context, CD9 has been shown to play an important role in the regulation of ALCAM-mediated cell adhesion and T cell activation. On leukocytes, CD9 acts as a scaffold in ALCAM-containing TEMs, engaging in direct lateral interactions through its LEL domain with the extracellular region of ALCAM, therefore, upregulating its adhesive capacity and co-stimulation in T cells through its ligand CD6 (29). Importantly, this enhancement of ALCAM avidity and functional activity induced by CD9 is mediated by a dual mechanism involving, on the one hand, augmented clustering of ALCAM molecules and, on the other hand, upregulation of ALCAM surface expression due to the inhibition of ADAM17/TACE sheddase activity (the latter will be discussed below) (Figure 1C). As CD9 has been shown to be part of TEMs that contain a number of proteins directly linked to ERM proteins (66), this tetraspanin could contribute an additional level of regulation to the dynamic association with the actin cytoskeleton that ultimately controls ALCAM avidity and costimulatory capacity.

## Endothelial CD9 Regulates Leukocyte Adhesion, Extravasation, and Inflammation

Migration across the endothelial cell layer that lines the inner surface of blood vessels is required for leukocyte recirculation during immune surveillance and their accumulation at sites of tissue infection and inflammation. Circulating leukocytes exit the blood vessels at certain preferred sites, such as the high endothelial venules of secondary lymphoid organs or the postcapil-lary venules at sites of inflammation. Leukocyte transendothelial migration proceeds through a series of steps which are mediated by a set of complex and sequential interactions between adhesion receptors and counter-receptors expressed on the leukocyte and the endothelial apical surface. This sequence of events represents the multi-step paradigm of the leukocyte extravasation cascade (79–81). These steps include the initial tethering and rolling of the leukocyte on the endothelial surface, followed by the firm adhesion, subsequent crawling on the endothelial apical surface in search for an appropriate exit site and the actual transmigration either between two endothelial cells (paracellular route) or through the body of an endothelial cell (transcellular route) and finally, leukocyte migration in the interstitial tissue following specific chemotactic cues. Specifically, the step of firm leukocyte adhesion to the endothelium is fundamentally mediated by the high-affinity interaction of leukocyte integrins, LFA-1 (αLβ2) and Mac-1 (αMβ2), with their endothelial counter-receptor ICAM-1, and of integrin VLA-4 (α4β1) with its endothelial ligand VCAM-1. These high-affinity interactions require the activation of integrins in response to intracellular signals triggered upon recognition by leukocyte chemokine receptors of chemokines immobilized on heparan sulfate proteoglycans on the endothelial apical surface.

Vascular endothelium plays an active role in transendothelial leukocyte migration by controlling the expression level and organization of adhesive molecules. Following exposure to proinflammatory cytokines (such as TNF-α and IL-1β), an increase in the expression of E- and P-selectins (main mediators of leukocyte tethering and rolling steps) and integrin ligands ICAM-1 and VCAM-1 is induced on the apical surface of endothelial cells, thus facilitating leukocyte adhesion to the endothelium and their subsequent transmigration. Interestingly, ICAM-1 and VCAM-1 on the endothelial surfaces are preorganized in adhesive clusters, so-called endothelial adhesive platforms (EAPs), by their inclusion in tetraspanin nanoplatforms through their association with CD9 and CD151 (24, 25). Analysis by FLIP-FRET microscopy evidenced that specificity exists in these associations, with CD9 preferentially associating with ICAM-1 whereas CD151 preferentially associates with VCAM-1 (24) (Figure 1B). Upon leukocyte binding, EAPs evolve into three-dimensional docking structures formed by elongated microvilli and long filopodia that emanate from the endothelial surface and embrace the adherent leukocytes, preventing their detachment under physiological hemodynamic flow conditions. The formation of these structures requires not only the tetraspanin-mediated clustering of ICAM-1 and VCAM-1, but also an active participation of the actin cytoskeleton and associated proteins, such as ERMs (ezrin–radixin–moesin), α-actinin, vinculin, filamin, cortactin, and vasodilator-stimulated phosphoprotein (VASP), as well as signaling molecules like Rho A/ROCK, MLCK, src, pyk-2, and PIP_2_ (82–84). Importantly, in the absence of CD9 the formation of microvilli required for the development of full docking structures is inhibited, crucially underlying the relevance of this particular tetraspanin in regulating the firm adhesion and transendothelial migration of leukocytes (85).

ALCAM is also abundantly expressed on endothelial cells in central nervous system (CNS) and has been shown to participate in the formation of docking structures (or transmigratory cups) required for leukocyte diapedesis (86). Interestingly, unlike ICAM-1 and VCAM-1, ALCAM mediates the transmigration of both lymphoid and myeloid leukocytes (86), and seems to be particularly relevant in the extravasation of monocytes rather than T cells across the BBB (87). Blockade of ALCAM reduced the transmigration of CD4^+^ lymphocytes and monocytes across the BBB and reduced the severity and delayed the time of onset of experimental autoimmune encephalomyelitis in animal models, highlighting the potential usefulness of ALCAM as a therapeutic target in multiple sclerosis (86). ALCAM has also been found to associate with tight junction molecules that maintain BBB integrity (88). The interaction of CD9 with ALCAM on endothelial cells has not been as yet properly explored, but considering that ALCAM participates in the formation of docking structures and what has been reported on leukocytic cells (29), it is likely that CD9 could also crucially regulate both the organization and the level of expression of ALCAM molecules on the CNS endothelium, critically controlling the transmigration of lymphocytes and monocytes across the BBB.

## CD9 Regulates the Shedding of Leukocyte Adhesion Molecules Mediated by ADAM10 and ADAM17 Metalloproteases

Another important mechanism by which CD9 is capable to influence the activity of adhesion molecules with relevance in the immune system is through the modulation of ADAM10 and ADAM17 metalloproteinases. These two closely related members of the a disintegrin and metalloproteinase (ADAM) family are key enzymes responsible for the cleavage and release from the cell surface of the ectodomains of a large variety of transmembrane proteins, a process known as *ectodomain shedding*. Through this process, cells can rapidly alter their surface phenotype by releasing a variety of soluble protein ectodomains that can subsequently trigger autocrine, juxtacrine, paracrine, or endocrine cellular signaling and responses in specific target cells. The list of identified protein substrates that can be shed either by ADAM10 or by ADAM17 has kept on growing over recent years and currently comprises over a hundred different cell surface proteins, including growth factors, cytokines, many types of receptors, and numerous cell adhesion molecules (CAMs). Among the CAMs shed by ADAM10 or ADAM17 there are members of the immunoglobulin (ICAM-1, L1-CAM, Ep-CAM, VCAM-1, ALCAM, and JAM-A), integrin, cadherin, and selectin families as well as other adhesion receptors like CD44 (89–91). Many of the protein substrates of these two proteases are shared, indicating that their function may sometimes be complementary or redundant, but the mechanisms that selectively regulate their individual activities and substrate preference are still poorly understood. Regulation of ADAMs’ shedding activities can be achieved at different levels, impacting upon their intracellular trafficking and maturation, metalloprotease activity, and accessibility of substrates [as reviewed in Ref. (92, 93)].

Tetraspanins are emerging as important regulators of ADAMs’ shedding activities. By dynamically regulating their inclusion in, or exclusion from specific TEMs, tetraspanins are shown to influence the relative localization of ADAMs and their substrates on the plasma membrane, thus exerting crucial regulatory effects on their sheddase activity [reviewed in Ref. (42, 94)].

CD9 and other tetraspanins (including CD53, CD81, CD82, and CD151), have been reported to associate with ADAM10 under mild solubilization conditions. Additionally, antibody engagement of CD81, CD82, or CD9 did indeed stimulate ADAM10-dependent shedding of its substrates TNF-α and EGF (43). However, subsequent experiments using more stringent detergent conditions indicated that these tetraspanins do not associate directly with ADAM10 (95). Such interactions, instead, seem to be mediated through a distinct subfamily of tetraspanins (TspanC8), whose specific and direct association with ADAM10 regulates its exit from the ER, enzymatic maturation, intracellular trafficking, subcellular localization, and its metalloprotease activity against different cell surface protein substrates (94, 96, 97).

Importantly, CD9 is the only tetraspanin that has so far been reported to associate directly with ADAM17 on the surface of leukocytes and endothelial cells. This direct association was demonstrated by image and biochemical techniques, including confocal microscopy, proximity ligation, co-immunoprecipitation, covalent crosslinking, and pull-down assays. Heterologous expression/overexpression of CD9 or treatment with agonist CD9-specific antibodies inhibited ADAM17-mediated shedding of different substrates from leukocytic cells, including TNF-α and the adhesion molecules ICAM-1 and ALCAM/CD166. Thus, while anti-CD81 mAbs enhanced the release of TNF-α mediated by ADAM10 (43), anti-CD9 mAbs regulated negatively the stimulated release of that proinflammatory cytokine mediated by the closely related sheddase ADAM17 (44). Additionally, CD9 knockdown increased ADAM17-mediated shedding of its substrates (29, 44). CD9-mediated inhibition of ALCAM shedding by ADAM17 resulted in increased ALCAM levels on the cell surface and augmented ALCAM-mediated cell adhesion (29) (Figure 1C). These findings were later confirmed for additional ADAM-17 substrates, like LR11 (SorLa), a member of the low density lipoprotein receptor family which binds ApoE and has a key role in cell migration, adhesion, and drug resistance (98).

## Concluding Remarks

CD9 is emerging as an important regulatory molecule that controls the expression and activity of different adhesion molecules of crucial importance in the immune system. At the IS, CD9 on the T cell side regulates the clustering and adhesive activity of integrins LFA-1 (αLβ1) and VLA-4 (α4β1), whereas on the APC side, CD9 regulates the expression of ALCAM/CD166 and the avidity of its interaction with CD6. Through the control exerted by CD9 on the expression and/or activity of these adhesive receptors, this tetraspanin regulates the stabilization of the IS and the subsequent activation of T lymphocytes.

On activated endothelial cells exposed to inflammatory cytokines, tetraspanins CD9 and CD151 play a crucial role in the step of firm leukocyte adhesion by arranging endothelial ICAM-1 and VCAM-1 in preorganized adhesive clusters, called EAPs. Upon leukocyte binding to the luminal endothelial surface, EAPs evolve into docking structures that embrace the leukocytes, preventing their detachment under flow conditions.

CD9 is the only tetraspanin that has been shown to associate directly with ADAM17 on leukocytes and endothelial cells. Through this direct interaction with ADAM17, CD9 inhibits the sheddase activity of this metalloproteinase against its substrates, thus regulating the balance between the membrane and soluble forms of crucial adhesion molecules, such as ICAM-1 and ALCAM, which are key in the processes of leukocyte extravasation and recruitment into inflamed tissues and stabilization of the IS.

These important regulatory effects exerted by CD9 on the activity of adhesion molecules with relevance in the immune system are summarized in the three panels of Figure 1.

## Author Contributions

CC conceived and wrote the manuscript. RR and CC designed the figures. BC, YM-P, and RR commented and edited the manuscript. All authors read and approved the final manuscript.

## Conflict of Interest Statement

The authors declare that the research was conducted in the absence of any commercial or financial relationships that could be construed as a potential conflict of interest.

## Abbreviations

ADAM, a disintegrin and metalloproteinase; ALCAM, activated leukocyte cell adhesion molecule; BBB, blood–brain barrier; CAMs, cell adhesion molecules; CNS, central nervous system; cSMAC, central supramolecular activation cluster; DCs, dendritic cells; EAPs, endothelial adhesive platforms; ER, endoplasmic reticulum; ICAM-1, intercellular adhesion molecule 1; Ig-SF, immunoglobulin superfamily; IL-2, interleukin-2; IS, immune synapse; LDL, low density lipoproteins; LFA-1, lymphocyte function-associated antigen 1; LPS, lipopolysaccharide; mAb, monoclonal antibody; MMP, matrix metalloproteinase; NK, natural killer cells; PBMCs, peripheral blood mononuclear cells; PHA, phytohemagglutinin; pSMAC, peripheral supramolecular activation cluster; TEMs, tetraspanin-enriched microdomains; TLR4, toll like receptor-4; TNF-α, tumor necrosis factor-alpha; VCAM-1, vascular cell adhesion molecule-1; VLA-4, very late activation antigen 4.

## References

[1] Boucheix C Rubinstein E. Tetraspanins. Cell Mol Life Sci (2001) 58:1189–205. 10.1007/PL00000933 11577978 PMC11337403

[2] Boucheix C Benoit P Frachet P Billard M Worthington RE Gagnon J Molecular cloning of the CD9 antigen. A new family of cell surface proteins. J Biol Chem (1991) 266:117–22. 1840589

[3] Boucheix C Benoit P CD9 antigen: will platelet physiology help to explain the function of a surface molecule during hemopoietic differentiation? Nouv Rev Fr Hematol (1988) 30:201–2. 2848215

[4] Levy S Shoham T. The tetraspanin web modulates immune-signalling complexes. Nat Rev Immunol (2005) 5:136–48. 10.1038/nri1548 15688041

[5] Hemler ME. Specific tetraspanin functions. J Cell Biol (2001) 155:1103–7. 10.1083/jcb.200108061 11756464 PMC2199333

[6] Hemler ME Tetraspanin proteins promote multiple cancer stages. Nat Rev Cancer (2013) 14:49–60. 10.1038/nrc3640 24505619

[7] Ovalle S Gutiérrez-López MD Olmo N Turnay J Lizarbe MA Majano P The tetraspanin CD9 inhibits the proliferation and tumorigenicity of human colon carcinoma cells. Int J Cancer (2007) 121:2140–52. 10.1002/ijc.22902 17582603

[8] Wright MD Moseley GW van Spriel AB. Tetraspanin microdomains in immune cell signalling and malignant disease. Tissue Antigens (2004) 64:533–42. 10.1111/j.1399-0039.2004.00321.x 15496196

[9] Hemler ME Targeting of tetraspanin proteins – potential benefits and strategies. Nat Rev Drug Discov (2008) 7:747–58. 10.1038/nrd2659 18758472 PMC4737550

[10] Charrin S le Naour F Silvie O Milhiet PE Boucheix C Rubinstein E. Lateral organization of membrane proteins: tetraspanins spin their web. Biochem J (2009) 420:133–54. 10.1042/BJ20082422 19426143

[11] Yanez-Mo M Barreiro O Gordon-Alonso M Sala-Valdes M Sanchez-Madrid F. Tetraspanin-enriched microdomains: a functional unit in cell plasma membranes. Trends Cell Biol (2009) 19:434–46. 10.1016/j.tcb.2009.06.004 19709882

[12] Lozahic S Christiansen D Manié S Gerlier D Billard M Boucheix C CD46 (membrane cofactor protein) associates with multiple beta1 integrins and tetraspans. Eur J Immunol (2000) 30:900–7. 10.1002/1521-4141(200003)30:3<900::AID-IMMU900>3.0.CO;2-X 10741407

[13] Jones PH Bishop LA Watt FM. Functional significance of CD9 associ-ation with beta 1 integrins in human epidermal keratinocytes. Cell Adhes Commun (1996) 4:297–305. 10.3109/15419069609010773 9117348

[14] Scherberich A Moog S Haan-Archipoff G Azorsa DO Lanza F Beretz A.Tetraspanin CD9 is associated with very late-acting integrins in human vas-cular smooth muscle cells and modulates collagen matrix reorganization. Arterioscler Thromb Vasc Biol (1998) 18:1691–7. 10.1161/01.ATV.18.11.1691 9812906

[15] Rubinstein E Le Naour F Lagaudrière-Gesbert C Billard M Conjeaud H Boucheix C. CD9, CD63, CD81, and CD82 are components of a surface tetraspan network connected to HLA-DR and VLA integrins. Eur J Immunol (1996) 26:2657–65. 10.1002/eji.1830261117 8921952

[16] Rubinstein E Le Naour F Billard M Prenant M Boucheix C. CD9 antigen is an accessory subunit of the VLA integrin complexes. Eur J Immunol (1994) 24:3005–13. 10.1002/eji.1830241213 7528664

[17] Berditchevski F Zutter MM Hemler ME. Characterization of novel complexes on the cell surface between integrins and proteins with 4 transmembrane domains (TM4 proteins). Mol Biol Cell (1996) 7:193–207. 10.1091/mbc.7.2.193 8688552 PMC275873

[18] Berditchevski F. Complexes of tetraspanins with integrins: more than meets the eye. J Cell Sci (2001) 114:4143–51. 11739647 10.1242/jcs.114.23.4143

[19] Indig FE Díaz-González F Ginsberg MH. Analysis of the tetraspanin CD9-integrin alphaIIbbeta3 (GPIIb-IIIa) complex in platelet membranes and transfected cells. Biochem J (1997) 327(Pt 1):291–8. 10.1042/bj3270291 9355765 PMC1218793

[20] Longhurst CM White MM Wilkinson DA Jennings LKA CD9, alphaIIbbeta3, integrin-associated protein, and GPIb/V/IX complex on the surface of human platelets is influenced by alphaIIbbeta3 conformational states. Eur J Biochem (1999) 263:104–11. 10.1046/j.1432-1327.1999.00467.x 10429193

[21] Reyes R Monjas A Yánez-Mó M Cardeñes B Morlino G Gilsanz A Different states of integrin LFA-1 aggregation are controlled through its association with tetraspanin CD9. Biochim Biophys Acta (2015) 1853(10 Pt A):2464–80. 10.1016/j.bbamcr.2015.05.018 26003300

[22] Shibagaki N Hanada K Yamashita H Shimada S Hamada H. Overexpression of CD82 on human T cells enhances LFA-1/ICAM-1-mediated cell-cell adhesion: functional association between CD82 and LFA-1 in T cell activation. Eur J Immunol (1999) 29:4081–91. 10.1002/(SICI)1521-4141(199912)29:12<4081::AID-IMMU4081>3.0.CO;2-I 10602019

[23] VanCompernolle SE Levy S Todd SC. Anti-CD81 activates LFA-1 on T cells and promotes T cell-B cell collaboration. Eur J Immunol (2001) 31:823–31. 10.1002/1521-4141(200103)31:3<823::AID-IMMU823>3.0.CO;2-D 11241287

[24] Barreiro O Zamai M Yáñez-Mó M Tejera E López-Romero P Monk PN Endothelial adhesion receptors are recruited to adherent leukocytes by inclusion in preformed tetraspanin nanoplatforms. J Cell Biol (2008) 183:527–42. 10.1083/jcb.200805076 18955551 PMC2575792

[25] Barreiro O Yáñez-Mó M Sala-Valdés M Gutiérrez-López MD Ovalle S Higginbottom A Endothelial tetraspanin microdomains regulate leukocyte firm adhesion during extravasation. Blood (2005) 105:2852–61. 10.1182/blood-2004-09-3606 15591117

[26] Schmidt DS Klingbeil P Schnolzer M Zoller M. CD44 variant isoforms associate with tetraspanins and EpCAM. Exp Cell Res (2004) 297:329–47. 10.1016/j.yexcr.2004.02.023 15212938

[27] Le Naour F Andre M Boucheix C Rubinstein E. Membrane microdomains and proteomics: lessons from tetraspanin microdomains and comparison with lipid rafts. Proteomics (2006) 6:6447–54. 10.1002/pmic.200600282 17109380

[28] Te Riet J Helenius J Strohmeyer N Cambi A Figdor CG Müller DJ. Dynamic coupling of ALCAM to the actin cortex strengthens cell adhesion to CD6. J Cell Sci (2014) 127:1595–606. 10.1242/jcs.141077 24496453

[29] Gilsanz A Sánchez-Martín L Gutiérrez-López MD Ovalle S Machado-Pineda Y Reyes R ALCAM/CD166 adhesive function is regulated by the tetraspanin CD9. Cell Mol Life Sci (2013) 70:475–93. 10.1007/s00018-012-1132-0 23052204 PMC11113661

[30] Slupsky JR Seehafer JG Tang SC Masellis-Smith A Shaw AR. Evidence that monoclonal antibodies against CD9 antigen induce specific association between CD9 and the platelet glycoprotein IIb-IIIa complex. J Biol Chem (1989) 264:12289–93. 2745443

[31] Boucheix C Duc GH Jasmin C Rubinstein E Tetraspanins and malignancy. Expert Rev Mol Med (2001) 3:1–17. 10.1017/S1462399401002381 14987371

[32] Tarrant JM Robb L van Spriel AB Wright MD. Tetraspanins: molecular organisers of the leukocyte surface. Trends Immunol (2003) 24:610–7. 10.1016/j.it.2003.09.011 14596886

[33] Toyo-oka K Yashiro-Ohtani Y Park CS Tai XG Miyake K Hamaoka T Association of a tetraspanin CD9 with CD5 on the T cell surface: role of particular transmembrane domains in the association. Int Immunol (1999) 11:2043–52. 10.1093/intimm/11.12.2043 10590270

[34] Horváth G Serru V Clay D Billard M Boucheix C Rubinstein E. CD19 is linked to the integrin-associated tetraspans CD9, CD81, and CD82. J Biol Chem (1998) 273:30537–43. 10.1074/jbc.273.46.30537 9804823

[35] Shi W Fan H Shum L Derynck R. The tetraspanin CD9 associates with transmembrane TGF-alpha and regulates TGF-alpha-induced EGF receptor activation and cell proliferation. J Cell Biol (2000) 148:591–602. 10.1083/jcb.148.3.591 10662783 PMC2174814

[36] Nakamura K Iwamoto R Mekada E. Membrane-anchored heparin-binding EGF-like growth factor (HB-EGF) and diphtheria toxin receptor-associated protein (DRAP27)/CD9 form a complex with integrin alpha 3 beta 1 at cell-cell contact sites. J Cell Biol (1995) 129:1691–705. 10.1083/jcb.129.6.1691 7790364 PMC2291180

[37] Anzai N Lee Y Youn BS Fukuda S Kim YJ Mantel C C-kit associated with the transmembrane 4 superfamily proteins constitutes a functionally distinct subunit in human hematopoietic progenitors. Blood (2002) 99:4413–21. 10.1182/blood.V99.12.4413 12036870

[38] Little KD Hemler ME Stipp CS. Dynamic regulation of a GPCR-tetraspanin-G protein complex on intact cells: central role of CD81 in facilitating GPR56-Galpha q/11 association. Mol Biol Cell (2004) 15:2375–87. 10.1091/mbc.E03-12-0886 15004227 PMC404030

[39] Yauch RL Hemler ME. Specific interactions among transmembrane 4 superfamily (TM4SF) proteins and phosphoinositide 4-kinase. Biochem J (2000) 351(Pt 3):629–37. 10.1042/bj3510629 11042117 PMC1221402

[40] Zhang XA Bontrager AL Stipp CS Kraeft SK Bazzoni G Chen LB Phosphorylation of a conserved integrin alpha 3 QPSXXE motif regulates signaling, motility, and cytoskeletal engagement. Mol Biol Cell (2001) 12:351–65. 10.1091/mbc.12.2.351 11179420 PMC30948

[41] Kaji K Oda S Shikano T Ohnuki T Uematsu Y Sakagami J The gamete fusion process is defective in eggs of Cd9-deficient mice. Nat Genet (2000) 24:279–82. 10.1038/73502 10700183

[42] Yanez-Mo M Gutierrez-Lopez MD Cabanas C. Functional interplay between tetraspanins and proteases. Cell Mol Life Sci (2011) 68:3323–35. 10.1007/s00018-011-0746-y 21687991 PMC11114976

[43] Arduise C Abache T Li L Billard M Chabanon A Ludwig A Tetras-panins regulate ADAM10-mediated cleavage of TNF-alpha and epidermal growth factor. J Immunol (2008) 181:7002–13. 10.4049/jimmunol.181.10.7002 18981120

[44] Gutiérrez-López MD Gilsanz A Yáñez-Mó M Ovalle S Lafuente EM Domínguez C The sheddase activity of ADAM17/TACE is regulated by the tetraspanin CD9. Cell Mol Life Sci (2011) 68:3275–92. 10.1007/s00018-011-0639-0 21365281 PMC11115118

[45] Miao WM Vasile E Lane WS Lawler J. CD36 associates with CD9 and integrins on human blood platelets. Blood (2001) 97:1689–96. 10.1182/blood.V97.6.1689 11238109

[46] Okamoto T Iwata S Yamazaki H Hatano R Komiya E Dang NH CD9 negatively regulates CD26 expression and inhibits CD26-mediated enhancement of invasive potential of malignant mesothelioma cells. PLoS One (2014) 9:e86671. 10.1371/journal.pone.0086671 24466195 PMC3900581

[47] Stipp CS Kolesnikova TV Hemler ME EWI-2 is a major CD9 and CD81 partner and member of a novel Ig protein subfamily. J Biol Chem (2001) 276:40545–54. 10.1074/jbc.M107338200 11504738

[48] Charrin S Le Naour F Oualid M Billard M Faure G Hanash SM The major CD9 and CD81 molecular partner. Identification and characterization of the complexes. J Biol Chem (2001) 276:14329–37. 10.1074/jbc.M011297200 11278880

[49] Pugholm LH Bæk R Søndergaard EK Revenfeld AL Jørgensen MM Varming K. Phenotyping of leukocytes and leukocyte-derived extracellular vesicles. J Immunol Res (2016) 2016:6391264. 10.1155/2016/6391264 27195303 PMC4852366

[50] Tohami T Drucker L Radnay J Shapira H Lishner M. Expression of tetraspanins in peripheral blood leukocytes: a comparison between normal and infectious conditions. Tissue Antigens (2004) 64:235–42. 10.1111/j.1399-0039.2004.00271.x 15304003

[51] Zuidscherwoude M Worah K van der Schaaf A Buschow SI van Spriel AB. Differential expression of tetraspanin superfamily members in dendritic cell subsets. PLoS One (2017) 12:e0184317. 10.1371/journal.pone.0184317 28880937 PMC5589240

[52] Gutierrez-Lopez MD Ovalle S Yanez-Mo M Sanchez-Sanchez N Rubinstein E Olmo N A functionally relevant conformational epitope on the CD9 tetraspanin depends on the association with activated beta1 integrin. J Biol Chem (2003) 278:208–18. 10.1074/jbc.M207805200 12411441

[53] Ovalle S Gutiérrez-López MD Monjas A Cabañas C Implication of the tetraspanin CD9 in the immune system and cancer. Inmunología (2007) 26:65–72. 10.1016/S0213-9626(07)70076-8

[54] Veenbergen S van Spriel AB Tetraspanins in the immune response against cancer. Immunol Lett (2011) 138:129–36. 10.1016/j.imlet.2011.03.010 21497620

[55] Grakoui A Bromley SK Sumen C Davis MM Shaw AS Allen PM The immunological synapse: a molecular machine controlling T cell activation. Science (1999) 285:221–7. 10.1126/science.285.5425.221 10398592

[56] Monks CR Freiberg BA Kupfer H Sciaky N Kupfer A Three-dimensional segregation of supramolecular activation clusters in T cells. Nature (1998) 395:82–6. 10.1038/25764 9738502

[57] Kim M Carman CV Springer TA. Bidirectional transmembrane signaling by cytoplasmic domain separation in integrins. Science (2003) 301:1720–5. 10.1126/science.1084174 14500982

[58] Kinashi T. Intracellular signalling controlling integrin activation in lymphocytes. Nat Rev Immunol (2005) 5:546–59. 10.1038/nri1646 15965491

[59] van Zanten TS Cambi A Koopman M Joosten B Figdor CG Garcia-Parajo MF. Hotspots of GPI-anchored proteins and integrin nanoclusters function as nucleation sites for cell adhesion. Proc Natl Acad Sci U S A (2009) 106:18557–62. 10.1073/pnas.0905217106 19850864 PMC2765922

[60] Bakker GJ Eich C Torreno-Pina JA Diez-Ahedo R Perez-Samper G van Zanten TS Lateral mobility of individual integrin nanoclusters orchestrates the onset for leukocyte adhesion. Proc Natl Acad Sci U S A (2012) 109:4869–74. 10.1073/pnas.1116425109 22411821 PMC3323969

[61] Lillemeier BF Mörtelmaier MA Forstner MB Huppa JB Groves JT Davis MM. TCR and Lat are expressed on separate protein islands on T cell membranes and concatenate during activation. Nat Immunol (2010) 11:90–6. 10.1038/ni.1832 20010844 PMC3273422

[62] Sherman E Barr V Manley S Patterson G Balagopalan L Akpan I Functional nanoscale organization of signaling molecules downstream of the T cell antigen receptor. Immunity (2011) 35:705–20. 10.1016/j.immuni.2011.10.004 22055681 PMC3225724

[63] Hogg N Patzak I Willenbrock F. The insider’s guide to leukocyte integrin signalling and function. Nat Rev Immunol (2011) 11:416–26. 10.1038/nri2986 21597477

[64] Rocha-Perugini V González-Granado JM Tejera E López-Martín S Yañez-Mó M Sánchez-Madrid F. Tetraspanins CD9 and CD151 at the immune synapse support T-cell integrin signaling. Eur J Immunol (2014) 44:1967–75. 10.1002/eji.201344235 24723389 PMC4630866

[65] van Kooyk Y Figdor CG. Avidity regulation of integrins: the driving force in leukocyte adhesion. Curr Opin Cell Biol (2000) 12:542–7. 10.1016/S0955-0674(00)00129-0 10978887

[66] Sala-Valdés M Ursa A Charrin S Rubinstein E Hemler ME Sánchez-Madrid F EWI-2 and EWI-F link the tetraspanin web to the actin cytoskeleton through their direct association with ezrin-radixin-moesin proteins. J Biol Chem (2006) 281:19665–75. 10.1074/jbc.M602116200 16690612

[67] Mittelbrunn M Molina A Escribese MM Yáñez-Mó M Escudero E Ursa A VLA-4 integrin concentrates at the peripheral supramolecular activation complex of the immune synapse and drives T helper 1 responses. Proc Natl Acad Sci U S A (2004) 101:11058–63. 10.1073/pnas.0307927101 15263094 PMC503740

[68] Hynes RO. Integrins: bidirectional, allosteric signaling machines. Cell (2002) 110:673–87. 10.1016/S0092-8674(02)00971-6 12297042

[69] Suzuki J Yamasaki S Wu J Koretzky GA Saito T. The actin cloud induced by LFA-1-mediated outside-in signals lowers the threshold for T-cell activation. Blood (2007) 109:168–75. 10.1182/blood-2005-12-020164 16973965

[70] Perez OD Mitchell D Jager GC South S Murriel C McBride J Leukocyte functional antigen 1 lowers T cell activation thresholds and signaling through cytohesin-1 and Jun-activating binding protein 1. Nat Immunol (2003) 4:1083–92. 10.1038/ni984 14528303

[71] Sato T Tachibana K Nojima Y D’Avirro N Morimoto C. Role of the VLA-4 molecule in T cell costimulation. Identification of the tyrosine phosphorylation pattern induced by the ligation of VLA-4. J Immunol (1995) 155:2938–47. 7673711

[72] Verma NK Kelleher D. Not just an adhesion molecule: LFA-1 contact tunes the T lymphocyte program. J Immunol (2017) 199:1213–21. 10.4049/jimmunol.1700495 28784685

[73] Mor A Dustin ML Philips MR. Small GTPases and LFA-1 reciprocally modulate adhesion and signaling. Immunol Rev (2007) 218:114–25. 10.1111/j.1600-065X.2007.00538.x 17624948

[74] Dustin ML. Cell adhesion molecules and actin cytoskeleton at immune synapses and kinapses. Curr Opin Cell Biol (2007) 19:529–33. 10.1016/j.ceb.2007.08.003 17923403 PMC2486492

[75] Zimmerman AW Joosten B Torensma R Parnes JR van Leeuwen FN Figdor CG. Long-term engagement of CD6 and ALCAM is essential for T-cell proliferation induced by dendritic cells. Blood (2006) 107:3212–20. 10.1182/blood-2005-09-3881 16352806

[76] Gimferrer I Calvo M Mittelbrunn M Farnós M Sarrias MR Enrich C Relevance of CD6-mediated interactions in T cell activation and prolife-ration. J Immunol (2004) 173:2262–70. 10.4049/jimmunol.173.4.2262 15294938

[77] Hassan NJ Barclay AN Brown MH. Frontline: optimal T cell activation requires the engagement of CD6 and CD166. Eur J Immunol (2004) 34:930–40. 10.1002/eji.200424856 15048703

[78] Tudor C te Riet J Eich C Harkes R Smisdom N Bouhuijzen Wenger J Syntenin-1 and ezrin proteins link activated leukocyte cell adhesion molecule to the actin cytoskeleton. J Biol Chem (2014) 289:13445–60. 10.1074/jbc.M113.546754 24662291 PMC4036352

[79] Butcher EC Leukocyte-endothelial cell recognition: three (or more) steps to specificity and diversity. Cell (1991) 67:1033–6. 10.1016/0092-8674(91)90279-8 1760836

[80] Springer TA Traffic signals for lymphocyte recirculation and leukocyte emigration: the multistep paradigm. Cell (1994) 76:301–14. 10.1016/0092-8674(94)90337-9 7507411

[81] Nourshargh S Alon R. Leukocyte migration into inflamed tissues. Immunity (2014) 41:694–707. 10.1016/j.immuni.2014.10.008 25517612

[82] Barreiro O Yanez-Mo M Serrador JM Montoya MC Vicente-Manzanares M Tejedor R Dynamic interaction of VCAM-1 and ICAM-1 with moesin and ezrin in a novel endothelial docking structure for adherent leukocytes. J Cell Biol (2002) 157:1233–45. 10.1083/jcb.200112126 12082081 PMC2173557

[83] Allingham MJ van Buul JD Burridge K. ICAM-1-mediated, Src- and Pyk2-dependent vascular endothelial cadherin tyrosine phosphorylation is requi-red for leukocyte transendothelial migration. J Immunol (2007) 179:4053–64. 10.4049/jimmunol.179.6.4053 17785844

[84] Heemskerk N van Rijssel J van Buul JD. Rho-GTPase signaling in leukocyte extravasation: an endothelial point of view. Cell Adh Migr (2014) 8:67–75. 10.4161/cam.28244 24621576 PMC4049863

[85] Franz J Brinkmann BF König M Hüve J Stock C Ebnet K Nanoscale imaging reveals a tetraspanin-CD9 coordinated elevation of endothelial ICAM-1 clusters. PLoS One (2016) 11:e0146598. 10.1371/journal.pone.0146598 26731655 PMC4701507

[86] Cayrol R Wosik K Berard JL Dodelet-Devillers A Ifergan I Kebir H Activated leukocyte cell adhesion molecule promotes leukocyte trafficking into the central nervous system. Nat Immunol (2008) 9:137–45. 10.1038/ni1551 18157132

[87] Lyck R Lécuyer MA Abadier M Wyss CB Matti C Rosito M ALCAM (CD166) is involved in extravasation of monocytes rather than T cells across the blood-brain barrier. J Cereb Blood Flow Metab (2017) 37:2894–909. 10.1177/0271678X16678639 28273717 PMC5536797

[88] Lécuyer MA Saint-Laurent O Bourbonnière L Larouche S Larochelle C Michel L Dual role of ALCAM in neuroinflammation and blood-brain barrier homeostasis. Proc Natl Acad Sci U S A (2017) 114:E524–33. 10.1073/pnas.1614336114 28069965 PMC5278491

[89] Scheller J Chalaris A Garbers C Rose-John S. ADAM17: a molecular switch to control inflammation and tissue regeneration. Trends Immunol (2011) 32:380–7. 10.1016/j.it.2011.05.005 21752713

[90] Reiss K Saftig P The “a disintegrin and metalloprotease” (ADAM) family of sheddases: physiological and cellular functions. Semin Cell Dev Biol (2009) 20:126–37. 10.1016/j.semcdb.2008.11.002 19049889

[91] Edwards DR Handsley MM Pennington CJ. The ADAM metalloprote-inases. Mol Aspects Med (2008) 29:258–89. 10.1016/j.mam.2008.08.001 18762209 PMC7112278

[92] Hartmann M Herrlich A Herrlich P. Who decides when to cleave an ectodomain? Trends Biochem Sci (2013) 38:111–20. 10.1016/j.tibs.2012.12.002 23298902

[93] Grotzinger J Lorenzen I Dusterhoft S. Molecular insights into the multilayered regulation of ADAM17: the role of the extracellular region. Biochim Biophys Acta (2017) 1864:2088–95. 10.1016/j.bbamcr.2017.05.024 28571693

[94] Seipold L Saftig P. The emerging role of tetraspanins in the proteolytic processing of the amyloid precursor protein. Front Mol Neurosci (2016) 9:149. 10.3389/fnmol.2016.00149 28066176 PMC5174118

[95] Dornier E Coumailleau F Ottavi JF Moretti J Boucheix C Mauduit P TspanC8 tetraspanins regulate ADAM10/Kuzbanian trafficking and promote Notch activation in flies and mammals. J Cell Biol (2012) 199:481–96. 10.1083/jcb.201201133 23091066 PMC3483123

[96] Saint-Pol J Eschenbrenner E Dornier E Boucheix C Charrin S Rubinstein E.Regulation of the trafficking and the function of the metalloprotease ADAM10 by tetraspanins. Biochem Soc Trans (2017) 45:937–44. 10.1042/BST20160296 28687716

[97] Matthews AL Noy PJ Reyat JS Tomlinson MG. Regulation of A disintegrin and metalloproteinase (ADAM) family sheddases ADAM10 and ADAM17: the emerging role of tetraspanins and rhomboids. Platelets (2017) 28:333–41. 10.1080/09537104.2016.1184751 27256961 PMC5490636

[98] Tsukamoto S Takeuchi M Kawaguchi T Togasaki E Yamazaki A Sugita Y Tetraspanin CD9 modulates ADAM17-mediated shedding of LR11 in leukocytes. Exp Mol Med (2014) 46:e89. 10.1038/emm.2013.161 24699135 PMC3944444

